# Role of Environmental Chemicals in Obesity: A Systematic Review on the Current Evidence

**DOI:** 10.1155/2013/896789

**Published:** 2013-06-05

**Authors:** Roya Kelishadi, Parinaz Poursafa, Fahimeh Jamshidi

**Affiliations:** ^1^Child Growth and Development Research Center, Isfahan University of Medical Sciences, Isfahan 81676-36954, Iran; ^2^Faculty of Medicine, Isfahan University of Medical Sciences, Isfahan 81676-36954, Iran; ^3^Environment Research Center, Isfahan University of Medical Sciences, Isfahan 81676-36954, Iran

## Abstract

The purpose of this paper is to systematically review the experimental and human studies on obesogenic chemicals and their mechanisms of action to provide a comprehensive view on the multifactorial aspects of obesity. The literatures were searched in available databases. The relevant papers were selected in three phases. After quality assessment, two reviewers extracted the data while another checked their extracted data. In this review, we summarized information regarding environmental chemicals that can be associated with obesity. Most evidence comes from experimental and laboratory studies; however a growing number of human studies also support the role of obesogenic chemicals. The current evidence proposes that the systemic responses to exposure to environmental factors could potentially increase the risk of excess weight. The effects of exposure to these chemicals are of crucial importance during developmental phases of life, when preprogramming for an adipogenic outcome may occur. By considering the adverse transgenerational effects of obesogen chemicals on human health, the global obesity epidemic should be considered as a multifactorial complex disorder necessitating the emphasis of public health interventions for environmental protection.

## 1. Introduction

Obesity is becoming a human health crisis at individual and public health levels. It has numerous adverse health effects and is considered as one of the main predisposing factors for the emerging epidemic of noncommunicable diseases [1]. Nowadays, overweight and obesity are growing in populations with different levels of economic situation. It is estimated that by continuing the actual trend, the global prevalence rate of 33.0% for overweight and obesity among adult population (1.3 billion people) in 2005 would reach up to 57.8% (3.3 billion people) by 2030 [2]. The World Health Organization included excess weight, with a prevalence higher than undernutrition, as one of the top 10 health risks worldwide [3].

The rise in the incidence in obesity matches the rise in the use and distribution of industrial chemicals that may have a role in development of obesity. In her interesting review in 2002, Baillie-Hamilton postulated a role for chemical toxins in the etiology of obesity by presenting the coincidence of the obesity epidemic with the noticeable increase of industrial chemicals in the environment over the past four decades. An accumulating body of evidence suggests that substances as endocrine-disrupting chemicals (EDCs) may be linked to the obesity epidemic [4]. EDCs are chemicals that alter the normal functioning of hormones and other signaling molecules in the body [5]. Additional studies proposed the existence of chemicals termed “obesogens,” molecules with adverse effects on lipid metabolism and adipogenesis, and in turn resulting in obesity [6, 7].

The environmental obesogen hypothesis suggests that prenatal or early-life exposure to certain substances as EDCs may predispose exposed individuals to increased fat mass and excess weight. It is suggested that exposure to obesogens can modify the epigenome of multipotent stromal stem cells, biasing them to the adipocyte lineage at the expense of bone. Hence, humans exposed to obesogens during early life might have an altered stem cell compartment, already preprogrammed for an adipogenic outcome [8]. 

The list of chemicals studied as possible obesogens continues to grow and includes diethylstilbestrol (DES), bisphenol A (BPA), phthalates, organotins, polybrominated diphenyl ethers (PBDEs), polyfluoroalkyl chemicals (PFCs), organochlorine (OC) pesticides, and polychlorinated biphenyls (PCBs) and some solvents caused weight gain, and it is proposed that these chemicals were interfering with weight homeostasis by changing weight-controlling hormones, modifying sensitivity to neurotransmitters, or altering the sympathetic nervous system activity [9].

The purpose of this paper is to systematically review the experimental and human studies on obesogenic chemicals and their mechanisms of action to provide a comprehensive view on underlying mechanisms and the multifactorial aspects of obesity for clinicians and public health stakeholders.

## 2. Methods

### 2.1. Literature Search Strategy

Relevant literature reporting the environmental obesogens was identified through electronic search of MEDLINE, PubMed, ISI Web of Science, and Scopus/Embase with no time or language restrictions. The literature search was conducted during January and February 2013. We searched the databases using the following strategy: for Scopus/Embase we used the *Emtree* Thesaurus terms; for PubMed search, we considered Medical Subheading (MeSH) words, and for other databases we used keywords (text words). For PubMed search, we used (“endocrine disruptors” [MeSH] OR “endocrine disrupting chemicals” OR “obesogen” [mh] OR “Polychlorinated Biphenyls” [MeSH] OR “Hydrocarbons, Chlorinated” [MeSH] OR “Dioxins” [MeSH] OR “Polybrominated Biphenyls” [MeSH] OR “Carbon Tetrachloride” [MeSH] OR “Organothiophosphorus Compounds” [MeSH] OR “phthalic acid” [Substance Name] OR “Phthalic Acids” [MeSH] OR “Organotin Compounds” [MeSH] OR “bisphenol A” [Substance Name] AND ((“obesity” [mh] OR “overweight” [mh] OR “excess weight” [mh] OR “body mass index” [mh] OR “weight gain” [mh] OR “adipogenesis” [mh] OR “adipose tissue” [mh] OR “fat deposition” [mh]) AND (publisher[sb] OR “in process” [sb]).

### 2.2. Study Selection and Eligibility Criteria

Duplicates were removed; the relevant papers were selected in three phases. In the first and second phases, titles and abstracts of papers were screened and irrelevant papers were excluded. In the last phase, the full text of recruited papers was explored intensely to select only relevant papers. For any additional pertinent studies, the reference list of all reviews and relevant papers was screened as well. All these three screening phases were done by two independent reviewers (FJ and PP). In the next step, the eligibility of relevant papers was checked. Discrepancies were resolved by consultation and consensus. 

### 2.3. Quality Assessment

Identification of main findings of studies was conducted on a case-by-case basis and included consideration of any statistical analyses that might have been conducted, consistency of the general pattern across exposure groups. 

### 2.4. Data Extraction and Abstraction

The required information that was extracted from all eligible papers was as follows: (i) general characteristics of the study (first author's name, publication year, study year, study design) (ii) characteristics of the chemical, (iii) reason for using the chemical, (iv) suggested obesogen mechanism, and (v) adverse effects on humans or animals.

Two reviewers (FJ and PP) extracted the data while another (RK) checked their extracted data. 

## 3. Results

**Figure 1:**
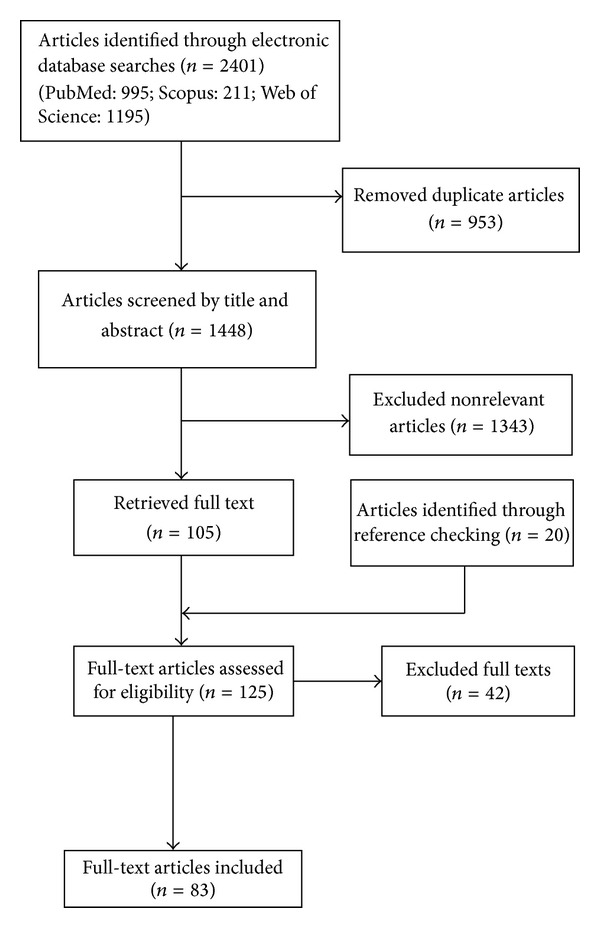
Flowchart of study selection.

The flowchart of our study selection is presented in Figure 1. We found that actually many environmental obesogens are identified; they are mainly classified as chemical simulators of metabolic hormones or brain neurotransmitters [10, 11]. Several experimental studies reported the association of exposure to some environmental chemicals with obesity. Bisphenol A [12–15], tributyltin (TBT) [16, 17], nonylphenol [18, 19] and genistein [20, 21], phatalate [22], perfluoroalkyl compounds (PFCs) [23], and perfluorooctanoic acid (PFOA) [24] are some of the obesogen chemicals described by experimental studies. The major environmental obesogen chemicals are presented in Table 1. 

Diverse mechanisms are explained for obesogen chemicals; mainly they have disruptive effects on homeostasis of energy balance, glucose and lipid metabolism, and control of adipogenesis. A summary of the underlying mechanisms of these substances is reported in Table 2.

Conflicting results are reported about the effects of obesogen chemicals in human studies. The concentrations of many industrial obesogen chemicals are found to be high in general population [25]. For instance, some studies examined the obesogenic effects of phthalates, which are esters mainly added to plastics to increase their flexibility, transparency, durability, and longevity. Cross-sectional data from the National Health and Nutrition Examination Survey (NHANES) in the USA found significant associations between several phthalates metabolites (monobenzyl phthalate (MBzP), mono-(2-ethyl-5-hydroxyhexyl) phthalate (MEHHP), and mono-(2-ethyl-5-oxohexyl) phthalate (MEOHP)) and measures of abdominal obesity and insulin resistance in men but not in women [26]. A cross-sectional study on 90 girls aged 6–8 years found slightly higher concentrations of some phthalate metabolites as monoethyl phthalate (MEP), mono-(2-ethyl-5-hydroxyhexyl) phthalate (MEHHP), and mono-n-butyl phthalate (MBP) among overweight girls than in their other counterparts; however the difference was not statistically significant [27]. Some epidemiologic studies documented the obesogenic effects of some environmental chemicals, as PCB [28] and BPA [29], whereas such effects are conflicting for some other chemicals as organochlorine pesticides [30–32].

Phytoestrogens, notably soy products, have beneficial health effects and are added to several food and food supplements. However some studies suggested that they may act as obesogen chemicals. Genistein is one the mostly used phytoestrogens in the human diet, and by its estrogenic activity, it has favorable effects for regulating the homeostasis of lipids and carbohydrates [33]. Though its beneficial effects in inhibiting fat deposition in the adipose tissue are considered to be obtained at high pharmacological doses, its low doses in foods are found to increase adiposity and mild peripheral insulin resistance particularly in males [34].

## 4. Discussion

In this review, we summarized information regarding environmental chemicals that can be associated with obesity. Most evidence comes from experimental and laboratory studies; however a growing number of human studies also support the role of obesogen chemicals. 

Chemicals as heavy metals, some solvents, pesticides, BPA, organophosphates, phthalates, PCB, PBBs, and many other substances are documented to cause weight gain. These chemicals interfere with weight and lipid homeostasis by various mechanisms related to weight-controlling hormones, activity of the sympathetic nervous system, and sensitivity to neurotransmitters. 

Exposure to these chemicals varies in different age groups; their effects during fetal and infancy periods may be irreversible and long-lasting for adulthood. Even exposure to low doses of EDCs during critical times of differentiation can change the developmental programming and may result in obesity [35]. Barker's hypothesis on the effects of intrauterine growth on fetal programming and fetal origins of adult diseases is well documented [36, 37]; however, other characteristics as later growth spurt and environmental factors are considered to influence this programming. Exposure to environmental chemicals with endocrine-disrupting activities in early life may result in everlasting adverse health effects [38]. Such health consequences may become apparent not only in childhood, but also in adulthood [5], and even in succeeding generations [39]. Transgenerational effects may be because of mutations as well as because of factors regulating gene expression [5]. Our findings support the role of obesogens, as chemicals with disruptive effects on fat homeostasis and various weight controlling mechanisms, in programming the development of excess weight from early life. Although all obesogen chemicals are not yet identified, and their detailed mechanisms of action remain to be explored, generally it is assumed that exposure to different doses of these environmental chemicals in various periods of life from fetal to adult period interacts with some endocrine signaling mechanisms and in turn leads to obesity.

EDCs act by diverse mechanisms; accumulating body of evidence supports that these chemicals disrupt some epigenetic, structural, and functional mechanisms, which control energy homeostasis, lipid metabolism, appetite regulation, and adipogenesis [40–44]. 

Chemical obesogens are considered to function through various factors as leptin, ghrelin, melanocyte-stimulating hormones, neuropeptide Y, amphetamine-regulated transcript, agouti-related protein, and cocaine, as well as through inhibiting aromatases as the P450 family members (CYP19 and CYP3A1) [42–44] or through modifying the expression of various receptors for steroid hormones, retinoic X, peroxisome proliferator-activated, and glucocorticoids [45]. The exposure to obesogen chemicals may influence the steroid hormone receptors or may change serum levels of metabolic hormones or may influence nuclear receptor signaling pathways in preadipocytes, which would result in adipocyte differentiation and a tendency to excess weight [44, 45].

The systemic reactions to exposure to environmental chemical factors can potentially increase the risk for obesity-related health effects, as metabolic syndrome, insulin resistance [46], prediabetes, diabetes, oxidative stress [47], prehypertension [48], hypertension, and nonalcoholic fatty liver diseases [49] even in the pediatric age group. 

Even in the pediatric age group, environmental chemicals can influence oxidative stress and proinflammatory cytokines [46, 47, 50], which in turn would initiate the second hit suggested in the “two-hit hypothesis” [51, 52] for the progression of fatty liver to metabolic syndrome and diabetes. 

The other aspect of the influences of environmental factors on obesity and its health consequences can be the impact of these chemicals on intrauterine growth retardation, low birth weight, and prematurity [53–55], which are documented as predisposing factors for obesity and adult chronic diseases.

Whether the results of laboratory models can be generalized to health hazards in humans remain to be determined, but a growing number of epidemiologic studies also suggest a link between exposure to environmental chemicals with obesity. However, it should be considered that in many human studies, weight gain has not been an endpoint in the original proposal, and excess weight has been reported as an adverse effect. 

Environmental factors have diverse health effects [47–50, 56–58]. Although rapid changes in lifestyle habits, along with increased energy intake and decreased energy expenditure, are considered as the main causes of excess weight, but by considering the rapid escalating trend of obesity in various age groups and in populations with different lifestyle habits and diverse socioeconomic levels, it is obvious that it is simple-minded to consider only these two factors responsible for this expanding global problem; the role of other environmental determinants as obesogen chemicals is being proposed in this regard. 

### 4.1. Study Limitations

Most studies included in this review have been observational and cross-sectional. Large-scale longitudinal studies with long-term followup are necessary to document the clinical importance of exposure to environmental chemicals. 

## 5. Conclusion

The current evidence proposes that the systemic responses to exposure to environmental factors, notably during developmental phases of life, could potentially increase the risk of excess weight. By taking into account the current knowledge on the adverse transgenerational effects of obesogen chemicals on human health, the global obesity epidemic should be considered as a multifactorial complex disorder necessitating the emphasis of public health interventions for environmental protection.

## Figures and Tables

**Table 1:** Summary of main obesogen chemicals and their health effects.

Chemicals	1st author	St.yr	Ge.Loc	Sample size	Study population	Characteristics	Uses	Mechanisms	Human effects	Animal effects
Phytoestrogens (genistein and daidzein)	Miriam J. J. de Kleijn [21] 2002	1971	USA	5209	30–59 y		Included in various food and food supplements, in particular soy product	High doses inhibited adipose deposition but at low doses similar to those found in Western and Eastern diets, in soy milk, or in food supplements containing soy, it induced adipose tissue deposition especially in males		

Perfluorooctanoic acid (PFOA)	Frank D. Gilliland [24]	1985–1989	USA	115				Perfluorooctanoic acid (PFOA) increases PPAR-dependent lipid mobilization, fatty acid oxidation, and adipose tissue atrophy during periods of experimental exposure. PFOA probably exerts anorexigenic effects through a central hypothalamic mechanism that triggers a decrease in food intake in adult rodents		

Perfluoroalkyl compounds (PFCs)	Sakr,CarineJ [23] 2007	2007		1025				Agonists for one or more of the PPARs, providing a mechanistic link to disturbed lipid and steroid metabolism		

Phtalates	Elizabeth E Hatch [22] 2008	1999–2002	USA	4369 participants	6–80 y		As plasticizers and stabilizers in a variety of plastics. They are found in industrial paints and solvents but also in cosmetics, perfumes, and medicines	PPAR*α*, PPAR*γ*, ER, and peptidergic hormones		

Nonylphenol	Mei-Lien Chen [19] 2009	2008	Taiwan	960	Primary and junior high schools					

Organotins, as tributyltin chloride (TBT) and bis (triphenyltin) oxide	Zhenghong Zuo [17] 2011	2009	China	32 mice	Mice, aged 21 days and weighing 10.5–13.5 g	Tetravalent tin compounds with a variety of mono-, di-, tri-, or tetrasubstituted organic functional groups	Antifouling agents in paints for marine shipping and for a variety of other uses	PPARg and RXR have been shown to disrupt normal development and homeostatic controls over adipogenesis and energy balance. suggested an inhibition of adipogenesis in the 3T3-L1 cells(1) TBT stimulates adipocytes differentiation in vitro and increases adipose mass in vivo in the 3T3-L1 cells		In-utero studies, showed TBT to accumulate lipids in adipose, testis, and liver tissues in neonate mice. and increasing epididymal adipose mass in adult mice

Bisphenol A (BPA)	He-xing Wang1 [15] 2012	2011	China	360	8–15 y	BPA is a small (228 Da) molecule which is used as a monomer in polymerization reaction to produce polycarbonate plastics	Used in food and water containers baby bottles, lining of food and beverage metal cans, medical tubing, epoxy resins, and dental fillings	BPA mimics the actions of E2 on blood glucose homeostasis via two pathways: a rapid pathway involving ncmER and a prolonged pathway involving ER. It inhibits adiponectin release and stimulates release of IL-6 and TNF*α*. mouse triggers 3T3-L1 cells (fibroblasts that can differentiate into adipocytes) to differentiate into adipocytes. Suppression of adiponectin and increased IL-6 and TNF*α*	There has been no information on BPA effects on human adipocytes	Mice treated with low doses of E2 or BPA showed rapid increases in insulin release and reduced plasma glucose. High dose for 15 d reduction in body weight. 3 m did not alter body weight and fat depot

**Table 2:** Summary of mechanisms suggested for main obesogen chemicals.

Mechanism	Acting by	Chemicals
Metabolic sensors	PPAR, RXR, TR	TBT, TPT, PFCs, phthalate
Sex steroid dysregulations	CYP19, ER, AR	TBT, TPT, phthalate, BPA, alkylphenol, phytoestrogen, DES
Central integration of energy balance	PH, HPA, EC, NE	TBT, TPT, phthalate, BPA, alkylphenol, phytoestrogen, SSRI, typical antidepressant, atypical antipsychotic
Metabolic point	GR signaling(11_HSD), HPT	TBT, TPT, PBDEs, Dithiocarbamates, Glycyrrhetinic acid,TZD

TBT: tributyltin; TPT: triphenyltin; BPA: bisphenol A; PFCs: perfluoroalkyl compounds; PBDEs: polybrominated diphenyl ethers; DES: diethylstilbestrol; SSRI: selective serotoninreuptake inhibitor; TZD: thiazolidinediones; NE: neuroendocrine effects; PH: peptidergic hormones; EC: endocannabinoid; HPT: hypothalamus-pituitary-thyroid; HPA: hypothalamus-pituitary-adrenal.; TR: thyroid hormone receptor; PPAR: peroxisome proliferator activated receptors; RXR: 9-cis retinoic acid receptor; ER: estrogen receptors; AR: androgen receptors.
